# Prognostic utility of Palliative Prognostic Index in advanced cancer: A systematic review and meta-analysis

**DOI:** 10.1017/S1478951525000021

**Published:** 2025-01-21

**Authors:** Si Qi Yoong, Hui Zhang, Dee Whitty, Wilson Wai San Tam, Wenru Wang, Davina Porock

**Affiliations:** 1 Duke-NUS Medical School, Singapore, Singapore; 2 Alice Lee Centre for Nursing Studies, Yong Loo Lin School of Medicine, National University of Singapore, Singapore, Singapore; 3 St. Andrew’s Community Hospital, Singapore, Singapore; 4 Centre for Research in Aged Care, Edith Cowan University, Joondalup, Western Australia, Australia; 5 Faculty of Public Health, Mahasarakham University, Kantharawichai, Thailand

**Keywords:** Palliative Prognostic Index, systematic review, advanced cancer, palliative care, end-of-life care

## Abstract

**Objectives:**

To evaluate the prognostic utility of Palliative Prognostic Index (PPI) scores in predicting the death of adults with advanced cancer.

**Methods:**

A systematic review and meta-analysis were conducted. Six databases were searched for articles published from inception till 16 February 2024. Observational studies reporting time-to-event outcomes of PPI scores used in any setting, timing and score cutoffs were eligible. Participants were adults with advanced cancer residing in any setting. Random effects meta-analysis was used to pool hazard, risk, or odds ratios. Findings were narratively synthesized when meta-analysis was not possible.

**Results:**

Twenty-three studies (*n* = 11,235 patients) were included. All meta-analyses found that higher PPI scores or risk categories were significantly associated with death and, similarly, in most narratively synthesized studies. PPI > 6 vs PPI ≤ 4 (pooled adjusted HR = 5.42, 95% confidence intervals [CI] 2.01–14.59, *p* = 0.0009; pooled unadjusted HR = 5.05, 95% CI 4.10–6.17, *p* < 0.00001), 4 < PPI ≤ 6 vs PPI ≤ 4 (pooled adjusted HR = 2.04, 95% CI 1.30–3.21, *p* = 0.002), PPI ≥ 6 vs PPI < 6 (pooled adjusted HR = 2.52, 95% CI 1.39–4.58, *p* = 0.005), PPI ≤ 4 vs PPI > 6 for predicting inpatient death (unadjusted RR = 3.48, 95% CI 2.46–4.91, *p* < 0.00001), and PPI as a continuous variable (pooled unadjusted HR = 1.30, 95% CI 1.22–1.38, *p* < 0.00001) were significant predictors for mortality. Changes in PPI scores may also be useful as a prognostic factor.

**Significance of results:**

A higher PPI score is likely an independent prognostic factor for an increased risk of death, but more research is needed to validate the risk groups as defined by the original development study. Meta-analysis results need to be interpreted cautiously, as only 2–4 studies were included in each analysis. Clinicians and researchers may find this useful for guiding decision-making regarding the suitability of curative and/or palliative treatments and clinical trial design.

## Introduction

Cancer patients and their families seek prognostic information to guide decision-making and emotionally prepare for end-of-life (Chu et al. 2020). Although physician survival prediction is widely utilized, it could be unreliable and unduly optimistic (Chu et al. 2020). To qualify for specialized care and guide treatment decisions, an accurate prognosis is necessary (Chu et al. 2019; Kutzko et al. 2022). Tools like the Palliative Prognostic Index (PPI) offer standardized estimates to address the limitations of clinician prediction. Other validated tools for advanced cancer patients include the Palliative Prognostic Score (Yoong et al. 2024), the Suprise Question (van Lummel et al. 2022), and the Prognosis in Palliative Care tool and the Objective Prognostic Score (Lee et al. 2021).

The European Association of Palliative Care (Maltoni et al. 2005) and the European Society for Medical Oncology identified the PPI as a key tool for predicting survival in advanced cancer patients (Stone et al. 2023). Developing using data from a Japanese inpatient hospice (Morita et al. 1999), the PPI score ranges from 0 to 15 and includes assessments of the Palliative Performance Scale, edema, dyspnea, and delirium (Morita et al. 2001), with higher scores indicating shorter survival.

The PPI has been validated in various cancer settings, such as hospices (Kim et al. 2014; Subramaniam et al. 2013), palliative care units (Gerber et al. 2021; Miyagi et al. 2021), community (Hamano et al. 2014), and hematology wards (Lee et al. 2022; Ohno et al. 2017). Palliative care nurses in the community hospitals easily integrated it into admission routines (Belanger et al. 2015). A web-based prognostic calculator that included PPI increased doctors’ confidence and willingness to discuss prognosis with patients and ability to tailor treatments according to prognosis (Hui et al. 2024). Additionally, healthcare professionals in aged care teams found it easy to use and not burdensome, with most recommending it to colleagues (Gerber et al. 2023). The PPI was particularly useful for uncertain prognoses, promoting end-of-life discussions and early recognition of dying. However, its challenges included distinguishing between acute and terminal delirium and when edema should be rated as present (Gerber et al. 2023).

The original study’s survival analysis divided patients into 3 groups: PPI ≤ 2, 2 < PPI ≤ 4, and PPI > 4. Log-rank analyses showed that PPI could differentiate survival across these groups (Morita et al. 1999). Validation studies typically presented log-rank tests and Kaplan–Meier curves but not hazard ratios (HR), odds ratios (OR), or risk ratios (RR). While Kaplan–Meier curves reveal crude survival differences among risk groups, they lack effect measures with 95% confidence intervals (CI) that adjust for other variables (Stel et al. 2011). Furthermore, validation studies did not always adhere to the original model’s risk group definitions, which may account for instances where survival differences were not significant (Palomar-Muñoz et al. 2018; Trejo-Ayala et al. 2018; Yoon et al. 2014).

The only review on the prognostic utility of PPI pooled HR and did not differentiate between adjusted and unadjusted effect sizes (Liu et al. 2018), making it difficult to confirm an independent association between PPI scores and survival. A previous review on prognostic tools, including PPI, also highlighted inconsistent reporting of HR and 95% CI among the studies, preventing a meta-analysis (Simmons et al. 2017). We previously conducted a meta-analysis evaluating the PPI’s performance in terms of discrimination and calibration for predicting cancer patients’ survival (Yoong et al. 2023). Building on the previous review’s findings, this systematic review and meta-analysis aimed to evaluate the utility of PPI as a prognostic tool for advanced cancer patients (i.e. locally advanced, metastatic, or incurable cancers). This review focuses on advanced cancer patients who face an increased need to plan for end-of-life decisions, including treatment, palliation and personal matters. Compared to other predictive tools, the PPI offers a simple, standardized assessment that is easy for clinicians to use without extensive training or complex technology. Its evidence-based scoring system ensures quick and effective assessments. The findings from this review aim to provide clinicians with the best information to support patients and their families.

## Methods

This review was reported according to the Preferred Reporting Items for Systematic Reviews and Meta-Analyses (PRISMA) guidelines (Table S1) (Page et al. 2021). Its protocol was registered in PROSPERO (CRD42023475009).

### Eligibility criteria

The inclusion criteria were as follows: (1) adults (≥18 years old) with advanced cancer of any type or those receiving palliative care; (2) studies reporting the association of PPI with death (HR, OR, RR, and 95% CI, including both adjusted or unadjusted effect sizes); (3) studies conducted in any setting, at any time and using any PPI cutoffs; (4) both prospective or retrospective studies (including peer-reviewed articles, dissertations/theses, and preprints); and (5) studies published in English, as the authors are fluent in English only.

Studies were excluded if they involved (1) adults without cancer (unless the noncancer participants were few, and ≥80% of the participants had cancer); (2) other versions of PPI, such as Functional PPI; (3) study designs other than those specified (e.g. experimental studies, reviews, letters to the editor); or (4) studies that only presented Kaplan–Meier curves, log-rank ratios or other descriptive analyses without reporting effect sizes.

### Search strategy

We searched PubMed, ScienceDirect, Embase, Web of Science, CINAHL, ProQuest, and Google Scholar for relevant articles published from inception to 16 February 2024 (Tables S2–S7) and reviewed the reference lists of relevant studies and reviews. First, we searched PubMed using keywords and Medical Subject Headings such as “palliative prognostic index,” “palliative care,” and “cancer.” Second, other databases were searched with similar terms. Finally, Google Scholar and ProQuest were used to locate grey literature. The initial search results were uploaded to Rayyan, and after removing duplicates, SQY and DW identified potential studies by reviewing titles and abstracts. They independently assessed full-text articles for eligibility, with any discrepancies resolved by HZ.

### Data extraction

Five studies were used to design and pilot test a standardized data extraction form. SQY extracted the data, which was then verified by DW and HZ. The extracted data included authors, country, study design, participant characteristics, and prognostic effect measures (e.g. HR, RR, OR). Any disagreements were resolved through discussion until a consensus was reached.

### Quality appraisal

The risk of bias was assessed independently by SQY and DP using the Quality in Prognosis Studies tool, which evaluates 6 domains: (1) study participation, (2) study attrition, (3) prognostic factor measurement, (4) outcome measurement, (5) study confounding, and (6) statistical analysis and reporting. Each domain was rated as high, moderate, or low risk of bias (Grooten et al. 2019; Hayden et al. 2013). A study was considered “low risk of bias” if all 6 domains, or 1 moderate domain, showed low bias. It was considered “high risk of bias” if at least 1 domain was rated high or 3 domains were rated moderate. Studies with intermediate ratings were classified as “moderate risk of bias” (Grooten et al. 2019). Any discrepancies were resolved through discussion. Figures were generated using robvis (McGuinness and Higgins 2021).

The modified Grading of Recommendations, Assessment, Development, and Evaluations (GRADE) framework for prognostic factor reviews was used to assess the overall certainty of evidence (Huguet et al. 2013). It evaluated 6 factors: investigation phase, study limitations, inconsistency, indirectness, imprecision, and publication bias. Evidence with a moderate or large effect size, or an exposure-response gradient, could lead to an upgrade in the quality of evidence (Huguet et al. 2013). Studies with Phase 3 explanatory outcomes were initially rated as high-quality evidence (Huguet et al. 2013; Kent et al. 2020). Outcomes based on at least 2 studies included in the meta-analyses were rated as high, moderate, low, or very low quality. Justifications were provided in the “Evidence Profile” tables using the GRADEproGDT software (GRADE handbook 2013; McMaster University and Evidence Prime Inc 2022).

### Data analysis

Meta-analysis was conducted using restricted maximum likelihood in JASP (version 0.19.1) (JASP Team 2024). Cochran’s *Q* test and *I*^2^ statistic were used to assess heterogeneity, with statistical significance set at *p* < 0.10. Heterogeneity was classified as unimportant (*I*^2^ = 0–40%), moderate (*I*^2^ = 30–60%), substantial (*I*^2^ = 50–90%), or considerable (*I*^2^ = 75–100%) (Higgins et al. 2019). Following Riley et al. (2019), we pooled adjusted and unadjusted effect measures, grouped similar categories of effect measures, and treated continuous effect measures separately. Extracted outcomes were standardized and reclassified into “high” versus “low” PPI risk groups, with effect sizes representing the risk of death as positive numbers. When meta-analysis was not feasible, results were summarized narratively.

## Results

### Search results

**Figure 1.**
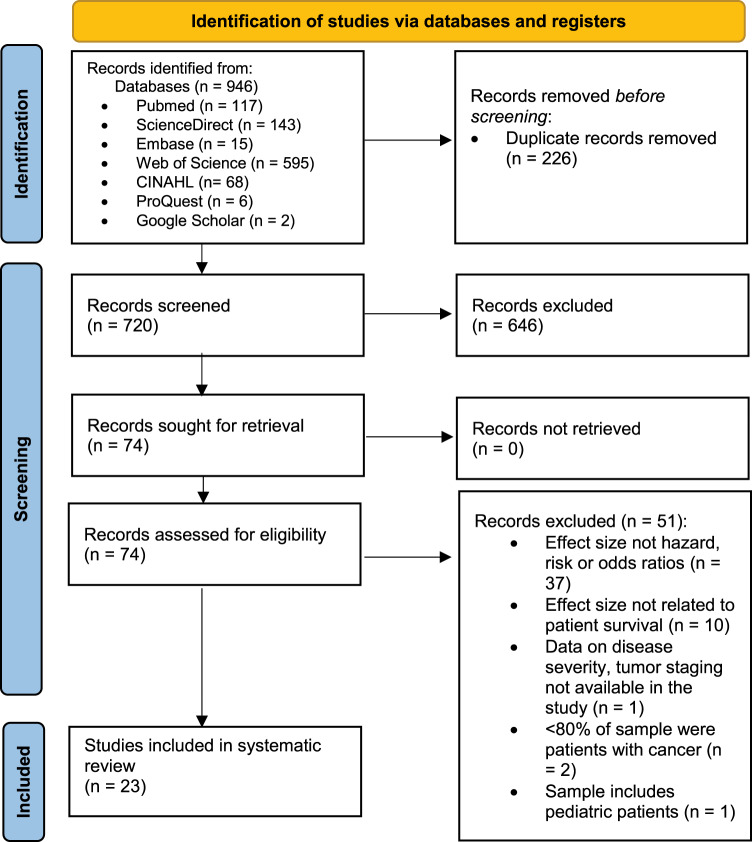
PRISMA diagram showing the study selection process.

Figure 1 illustrates the study selection process. The initial search identified 946 records. After removing duplicates, 720 records were screened by title and abstract, and 74 articles were further assessed through full-texts review. Ultimately, 23 articles from 21 patient cohorts were included in this systematic review. Reasons for exclusion are detailed in Table S8.

### Characteristics of included studies

Characteristics of the included studies are presented in Table 1. Studies were published between 2008 and 2023, using prospective (*n* = 9) (Chen et al. 2018; Fernandes et al. 2021; Hung et al. 2014; Kao et al. 2014; Lee et al. 2014; Miura et al. 2015; Palomar-Muñoz et al. 2018; Stone et al. 2008; Subramaniam et al. 2013) or retrospective designs (*n* = 14) (Ahn et al. 2021, 2016; Al-Ansari et al. 2022; Arai et al. 2014; Arkın and Aras 2021; Chang et al. 2021; Cheng et al. 2012; Chou et al. 2015; Gerber et al. 2021; Iizuka-Honma et al. 2023; Inomata et al. 2014; Kiuchi et al. 2022; Shatri et al. 2021; Trejo-Ayala et al. 2018).

**Table 1.** Characteristics of included studies

				Participants				
Author (year) Country	Study design	Settings	Period of participant recruitment and follow-up	Diagnoses (primary cancer site)	Under palliative care?	Receiving anticancer treatment?	Sample size (Male/Female)	Age
Ahn et al. (2016) Korea	Retrospective cohort study	Palliative care unit	January to December 2014 Until death	Terminal cancer – expected survival less than 3−6 months – esophagus/stomach, colorectal, liver, pancreas/biliary duct, lung, breast, breast, urogenital, and others	✓	?	205 (113/92) – 196 with PPI scores	- 65 years old (*n* = 117) –≤65 years old (*n* = 88)
Ahn et al. (2021) Korea	Retrospective chart audit	Hospice care unit patients with terminal cancer who received continuous deep sedation	January2014 to December 2016	Terminal cancer - hepato-biliary-pancreatic, gastrointestinal (esophagus, stomach, colorectal), lung, genitourinary (bladder, prostate, cervix, ovary, endometrial), breast, and others	✓	✘	106 (71/35)	Mean ± SD = 65.2 ± 12.8
Al-Ansari et al. (2022) Kuwait	Retrospective cohort study	Patients referred to palliative care center	1 January 2015 to 31 December 2018 Follow-up not reported	Advanced cancer not fit for further treatment - gastrointestinal, breast, thoracic, head and neck, hematologic, and others	✓	✘	719 (342/377)	Mean ± SD = 62.97 ± 13.65
Arai et al. (2014) Japan	Retrospective cohort study	Palliative care unit – those hospitalized for less than 6 days excluded	1 May 2007 to 31 July 2010 Death (within 3 weeks)	Terminal cancer	✓	None received radical antineoplastic therapy as curative therapy Some received radiation for pain relief	374 (208/166)	Median (range) = 64 (56−71)
Ahn et al. (2021) Turkey	Retrospective study	Palliative care clinic in a hospital	15 November 2016 to 15 April 2018 Follow-up till 3 months after end of study. Date of death obtained from patient files (inpatient deaths) or from National Death Reporting System (outpatient deaths)	Advanced lung cancer	✓	✘	203 (177/26)	Mean ± SD = 64.59 ± 10.87
Chang et al. (2021) Taiwan	Retrospective case control study (preprint)	First palliative consultation for patients receiving hospice care	January to August 2017	Hematological malignancies	✓	?	53 (40/13)	Mean (range) = 76.7 (69−83)
Chen et al. (2018) Brazil	Prospective observational study	Cancer center – Inpatients evaluated for palliative radiotherapy	November 2014 to December 2015 Until death or last follow-up	Lung, breast, prostate, stomach, head and neck, rectum, esophagus, kidney, and others	✓	77/333 receiving palliative chemotherapy 271/333 offered radiotherapy, 213 completed treatment	333–320 included in analysis	[Median (range)] = 58 (18−87)
Cheng et al. (2012) Taiwan	Retrospective study – used by nurse specialists	Referred to hospice consultation service	1 January to 30 June 2011 Until death, transfer to inpatient hospice, home hospice or discharge from hospital under stable condition	Metastatic or locally advanced cancer with less than 6 months to live – those referred from emergency rooms or undergoing cancer treatments of curative intent excluded – lung, liver, colorectal, upper gastrointestinal tract cancer, head and neck cancer, breast cancer, and others	✓	✘	623 (378/245)	Median (IQR) = 62 (52−73)
Chou et al. (2015) **–** Same cohort as Kao et al. (2014) and Hung et al. (2014) Taiwan	Retrospective cohort study – used by palliative car e physician or nurse specialist	Palliative care consultation service in a medical center	January 2006 to December 2011 Until death or for 180 days from first day of referral, whichever earlier – outpatients: death via institutional cancer registration center or National Register of Death Database	Hematologic malignancy – acute leukemia – lymphoma – multiple myeloma	✓	?	4685–217 (133/84) included in analysis	Median (IQR) = 63.3 (46−76)
Fernandes et al. (2021) Brazil	Prospective cohort study	Palliative care service at a tertiary hospital	May 2011 to December 2018 Follow-up until death, decline in participation in palliative care program or transfer to other hospitals	Any primary site tumor, including both hematologic and solid malignant neoplasms – those with potentially curable disease were excluded	✓	✘	1381 (672/709) – 1376 included in analysis	Mean (range) = 68 (21−100)
Gerber et al. (2021) Australia	Retrospective study	Patients in palliative care inpatient ward who were referred and seen by hospital’s Aged Care Assessment Team for residential aged care placement	2014 to 2015 Follow-up not reported	Malignant (57/71) and nonmalignant (14/71)	✓	✘	71 (32/39)	Mean ± SD = 80.25 (10.35)
Hung et al. (2014) **–** same cohort as Kao et al. (2014), Chou et al. (2015) Taiwan	Prospective cohort study – used by palliative car e physician or nurse specialist	Palliative care consultation service in a hospital	January 2006 to December 2011 Until death or for 180 days from first day of referral, whichever earlier – outpatients: death via institutional cancer registration center or National Register of Death Database	Terminal cancer – gastrointestinal tract, thoracic, head and neck, genitourinary tract, breast, and others – initial PPI > 6 Excluded – those died within 1 week after consultation – discharged from hospital or transfer from hospice ward within 1 week after referral	✓(3 doctors specialized in palliative care and family medicine)	?	4685–1035 included in analysis (634/401)	Median = 60.3
Iizuka-Honma et al. (2023) Japan	Retrospective study	Patients receiving end-of-life chemotherapy during last hospitalization	May 2015 and May 2021 Patients who died at the hospital during this period	Hematological malignancies (247/265) – malignant lymphoma (n = 113), leukemia (*n* = 93), multiple myeloma (*n* = 41)	?	✓ – 82 received chemotherapy (57 received in last 30 days of life)	265 (147/118)	Mean = 73.2
Inomata et al. (2014) Japan	Retrospective study	Admitted to hospital to receive palliative care	Patients who died between 2009 and 2013	Lung cancer Excluded: – history of anticancer therapy, e.g. surgery, radical irradiation, brain irradiation, chemotherapy after admission – absence of detection of recurrence after radical surgery or therapy – admission to hospital mainly for acute illness, e.g. infection, adverse effects of treatment – death from other causes than lung cancer	✓	Included: Receiving palliative irradiation	84 (74/10)	Median (range) = 72.5 (45−88)
Kao et al. (2014) **–** Same cohort as Hung et al. (2014), Chou et al. (2015) Taiwan	Prospective cohort study – used by palliative car e physician or nurse specialist	Palliative care consultation service in a hospital	January 2006 to December 2011 Until death or for 180 days from first day of referral, whichever earlier – outpatients: death via institutional cancer registration center or National Register of Death Database	Terminal cancer – head and neck, thoracic, breast, gastrointestinal, genitourinary, and others	✓	?	4685–2392 included in analysis (1429/963)	Median = 58.7
Kiuchi et al. (2022) Japan	Retrospective study	Patients receiving palliative chemotherapy in hospital	2014 to 2019 Those who died from ovarian cancer in hospital	Ovarian cancer (chemotherapy-resistant and chemotherapy-refractory patients)	✓	✓- Palliative chemotherapy	36	Median (range) at death = 57 (19−80)
Lee et al. (2014) Korea	Prospective observational study	Hospice inpatients	1 January 2013 to 31 May 2013	Terminal cancer (digestive tract, lung, hematology, bladder/renal and others) Excluded: – Unable to communicate (deterioration, severe cognitive impairment) – Lymphedema of upper or lower extremity – History of edema (heart failure, liver failure, inferior or superior vena cava syndrome) – Use of diuretics in medical record – Patients with ascites, pleural effusions and pericardial effusion	✓	?	28 (13/15)	≤50 (n = 3) 51−70 (n = 10) > 70 (n = 15)
Miura et al. (2015) Japan	Multicenter prospective cohort study	16 palliative care units, 19 hospital-based palliative care teams, 23 home-based palliative care services	September 2012 to April 2014	Locally extensive or metastatic cancer and newly referred to palliative care units, hospital-based palliative care teams, 23 home-based palliative care services	✓	✘	1160 (677/483)	Median (IQR) = 72 (63−80)
Palomar-Muñoz et al. (2018)Spain	Prospective cohort study	Palliative care unit	December 2013 – December 2015 Until death or end of study (15 February 2016) by mobile palliative team	− 165/322 hospitalized due to acute concomitant diseases e.g. infections, acute hemorrhage, cardiovascular acute syndromes, pulmonary thromboembolism, and others – 157/322 hospitalized without acute concomitant diseases, e.g. refractory symptoms, disease progression, exhaustion Cancer – respiratory, gastrointestinal, liver, bile duct, pancreas, genitourinary, breast, central nervous system, and others	✓	✘– none received palliative radiotherapy and/or chemotherapy	322 (196/126)	Mean ± SD = 71 ± 13
Shatri et al. (2021) Indonesia	Retrospective cohort study	Consult to a palliative care team at tertiary hospital	July 2017 to December 2018 Follow-up not reported	Advanced cancer – gynecology, breast, head and neck, digestive, Hepatocellular carcinoma, lung, skin and soft tissue, urology, bone, hematology	✓	?	160 (51/109)	Mean = 50.08
Stone et al. (2008) Ireland	Prospective study – recorded by nurse specialist or doctor	Specialist palliative care service – consultancy service based in hospital - community-based hospice home care service - 6-bedded hospice inpatient unit	Period of participant recruitment not reported Follow-up till death by obtaining information from the facilities or from death notices	Incurable cancer – bronchial carcinoma, colorectal, breast, hematological, prostate/bladder/kidney, pancreas/heptobiliary, gynecological, upper GI, and others	✓	60 having chemotherapy 36 having radiotherapy 13 having both	194 (100/94) – 151 with actual survival data	Mean ± SD = 69.9
Subramaniam et al. (2013)UK	Multicenter prospective study	3 inpatient hospices	January to June 2009 Until death or for 6 weeks after recruitment of last inpatient of each hospice (both inpatient and discharged), whichever earlier. Community palliative care teams recorded date of death by checking with patients’ general practitioner	Cancer	✓	?	265–262 included in analysis (134/128)	Median (range) = 71.7 (23−98)
Trejo-Ayala et al. (2018) Mexico	Retrospective cohort study	Hospital	2013 and 2014	Acute lymphoblastic leukemia	✓	✓ – Low intensity palliative care = CVBP chemotherapy±GCSF	32 (18/14)	Mean (range) = 37 (18−75)

SD = standard deviation; IQR = interquartile range;, ✓ = Yes; ? = unclear; ✘ = No.

The majority of studies were conducted in Asia and Australia (*n* = 17) (Ahn et al. 2021, 2016; Al-Ansari et al. 2022; Arai et al. 2014; Arkın and Aras 2021; Chang et al. 2021; Cheng et al. 2012; Chou et al. 2015; Gerber et al. 2021; Hung et al. 2014; Iizuka-Honma et al. 2023; Inomata et al. 2014; Kao et al. 2014; Kiuchi et al. 2022; Lee et al. 2014; Miura et al. 2015; Shatri et al. 2021), followed by Europe (*n* = 3) (Palomar-Muñoz et al. 2018; Stone et al. 2008; Subramaniam et al. 2013) and the Americas (*n* = 3) (Chen et al. 2018; Fernandes et al. 2021; Trejo-Ayala et al. 2018).

The review included 11,235 patients aged 18–100, with sample sizes ranging from 28 to 4,685. Most studies involved a mix of primary cancers, while 7 studies focused on a single cancer type (Arkın and Aras 2021; Chang et al. 2021; Chou et al. 2015; Iizuka-Honma et al. 2023; Inomata et al. 2014; Kiuchi et al. 2022; Trejo-Ayala et al. 2018). The majority of studies were conducted in palliative care settings, with 1 conducted in acute wards (Iizuka-Honma et al. 2023).

Twenty studies reported HR, with 12 adjusting for covariates (Ahn et al. 2021, 2016; Arai et al. 2014; Chang et al. 2021; Chou et al. 2015; Hung et al. 2014; Inomata et al. 2014; Kao et al. 2014; Kiuchi et al. 2022; Lee et al. 2014; Miura et al. 2015; Palomar-Muñoz et al. 2018). Most studies treated PPI as a categorical variable, while 5 analyzed it as a continuous variable (Arai et al. 2014; Gerber et al. 2021; Lee et al. 2014; Stone et al. 2008; Subramaniam et al. 2013). Dichotomous outcomes were extracted or computed from 5 studies (Al-Ansari et al. 2022; Arkın and Aras 2021; Fernandes et al. 2021; Gerber et al. 2021; Trejo-Ayala et al. 2018), with 2 reporting adjusted effect sizes (Al-Ansari et al. 2022; Gerber et al. 2021). The findings from each study are presented in Table 2.

**Table 2.** Prognostic effect measures reported in the included studies

Author (year)	Risk groups	Statistical analyses	Significant difference in survival between groups	PPI as continuous/ categorical variables	Unadjusted HR (95% CI)	Adjusted HR (95% CI)	RR/OR (95% CI)	Covariates of adjusted models
Ahn et al. (2016)	PPI ≤ 6 PPI > 6	Kaplan–Meier curve, log-rank test HR	Yes	Categorical	1.68 (1.26−2.25)	1.59 (1.16−2.17)	–	Male, poor performance measured by ECOG PS, current infection, thrombocytopenia, azotemia, hypoalbuminemia, hyperbilirubinemia, high CRP level, high NLR level
Ahn et al. (2021)	PP > 6 PPI ≤ 6	Median survival and 95% CI HR	Yes	Categorical	–	– Age and sex-adjusted: 1.68 (95% CI 1.10−2.56) – Backward multivariate (included significant variables in univariate analyses): 2.18 (95% CI 1.29−3.66)	–	– Age and sex – ECOG-PS = 4, hyperbilirubinemia, high ferritin, number of continuous deep sedation drugs
Al-Ansari et al. (2022)	Not reported	OR	Not applicable	–	–	–	Linear regression of PPI score on admission (higher PPI scores on admission were independent negative predictors of length of stay – Adjusted OR =−4.429 (−5.460 to −3.398)	Not reported
Arai et al. (2014)	PPI ≤ 4 4 < PPI ≤ 6 PPI > 6 PPI change per day = (5^th^ to 7^th^ day PPI – initial PPI)/day	Kaplan–Meier curve, log-rank test HR	Yes	Continuous	Initial PPI in predicting death within 3 weeks – 1.2 (1.2−1.3) Change in PPI in predicting death within 3 weeks – 4.9 (3.8−6.3)	Initial PPI in predicting death within 3 weeks – 1.3 (1.2−1.4) – with initial PPI ≤ 4: 1.4 (1.1−1.8) – with 4 < initialPPI ≤ 6: 2.9 (1.3−6.3) – with PPI>6: 1.4 (1.1−1.7)Change in PPI in predicting death within 3 weeks – 6.6 (4.9−9.0) – with initial PPI ≤ 4: 9.3 (5.8−15.0) – with 4 < initial PPI ≤ 6: 14.4 (5.7−36.2) – with PPI > 6: 9.1 (4.1−20.0)	–	Gender, age, BMI, body temperature, systolic and diastolic blood pressure, pulse rate, initial and change in PPI
Ahn et al. (2021)	PPI ≤ 4 4 < PPI ≤ 6 PPI > 6	Kaplan–Meier curve, log-rank test RR	Yes	Categorical	–	–	(RR calculated from data reported)	–
Chang et al. (2021)	PPI ≤ 6 PPI > 6	HR	Yes	Categorical	2.26 (0.94−5.44)	2.82 (1.32−6.03)	–	Disease type, age > 65, gender, PPI score >6, aggressive interventions including blood transfusion, antibiotic use, oxygen supplementation, and pain control
Chen et al. (2018)	(A) PPI ≤ 2 (B)2 < PPI ≤ 4 (C) PPI > 4	Kaplan–Meier curve, log-rank test HR	Yes	Categorical	– B vs A: 1.84 (1.07−3.16) – C vs A: 3.45 (2.07−5.74)	–	–	–
Cheng et al. (2012)	(A) PPI ≤ 4 (B) 4 < PPI ≤ 6 (C) PPI > 6	Kaplan–Meier curve, log-rank test HR	Yes	Categorical	A vs C: 0.19 (0.10−0.24) B vs C: 0.54 (0.43−0.69)	–	–	–
Chou et al. (2015)	(A) PPI ≤ 4 (B) 4 < PPI ≤ 6 (C) PPI > 6	Kaplan–Meier curve, log-rank test HR	Not reported	Categorical	–	– A vs B: 1.73 (1.07−2.80) – A vs C: 3.40 (2.31−4.98)	–	Age, gender, type of hematologic malignancy
Fernandes et al. (2021)	PPI ≤ 4 4 < PPI ≤ 6 PPI > 6	Kaplan–Meier curve, log-rank test RR	Yes	Categorical	–	–	RR: ≥ 6 vs < 6- to 3-week mortality: 6.11 (4.54−8.23) – 6-week mortality: 5.44 (3.72−7.96) RR: ≥ 4 vs < 4 – 3-week mortality: 6.68 (4.44−7.38) – 6-week mortality: 5.61 (3.99−7.89)	–
Gerber et al. (2021)	PPI > 6 PPI ≤ 4	RR (calculated from data provided) OR	Not applicable	Both	–	–	Continuous variable – unadjusted OR 0.74 (0.61−0.91) – adjusted OR (not significant) Categorical (<3 weeks vs > 6 weeks): – unadjusted OR 9.93 (2.72−36.29) – adjusted OR 12.36 (2.81−54.4)	OR: Malignancy, absence of anorexia, normal oral intake amount, Karnofsky Performance Scale, mPaP score
Hung et al. (2014)	(A) <−20% change (B) − 20 to 0% change (C) 0 change (D) 0−20% change (E) > 20% change Change in PPI = (8th day PPI – initial PPI)/(15-initial PPI) × 100	Kaplan–Meier curve, log-rank test HR	Not reported	Categorical	–	– A vs E: 0.17 (0.13−0.23) – B vs E: 0.32 (0.24−0.44) – C vs E: 0.49 (0.41−0.58) – D vs E: 0.83 (0.67−1.03)	–	Gender, age, primary cancer origin, referring medical department, interval between hospital admission and palliative care consultation service
Iizuka-Honma et al. (2023)	(A) PPI ≤ 2 (B) 2 < PPI ≤ 4 (C) PPI > 4	Kaplan–Meier curve, log-rank test HR	Yes	Categorical	B and C vs A: 2.1290 (95% CI 1.1830−3.828)	–	–	Not reported
Inomata et al. (2014)	PPI ≤ 6 PPI > 6	Kaplan–Meier curve, log-rank test HR	Yes	Categorical	–	4.06 (2.35−6.92)	–	Gender, age, histological diagnosis, EGFR gene status
Kao et al. (2014)	Initial PPI (A) PPI ≤ 2 (B) 2 < PPI ≤ 4 (C) PPI > 4 Week 1 PPI (A) PPI ≤ 2 (B) 2 < PPI ≤ 4 (C) PPI > 4 Change in PPI scores between initial and Week 1 (D) > 0 (E) 0 (F) < 0 Change in PPI = Initial PPI – 8th day PPI	Kaplan–Meier curve, log-rank test HR	Yes	Categorical	–	Initial PPI – A vs C: 0.43 (0.31−0.51) – B vs C: 0.61 (0.56−0.69) Week 1 PPI – A vs C: 0.33 (0.28−0.39) – B vs C: 0.47 (0.42−0.53) Change in score – D vs F: 0.47 (0.41−0.53) – E vs F: 0.63 (0.57−0.69)	–	Gender, age, primary cancer origin, referring medical department, interval between hospital admission and palliative care consultation service
Kiuchi et al. (2022)	PPI ≤ 4 PPI > 4	Kaplan–Meier curve, log-rank test HR	Yes	Categorical	2.91 (1.41−6.00)	1.76 (0.81−3.87)	–	Not reported
Lee et al. (2014)	Not applicable	HR	Not applicable	Continuous	1.21 (1.00−1.47)	1.23 (0.97−1.57)	–	Age, PPI, BMI
Miura et al. (2015)	(A) PPI < 4 (B) 4 ≤ PPI < 6 (C) PPI ≥ 6	HR	Not applicable	Categorical	– B vs A: 1.00 (0.86−1.16) – C vs A: 2.08 (1.83−2.36)	– B vs A: 1.11 (0.89−1.38) – C vs A: 1.56 (.27−1.92)	–	Primary tumor site, age, gender
Palomar-Munoz et al. (2018)	(A) PPI ≤ 4 (B) 4 < PPI ≤ 6 (C) PPI > 6 (D) with acute concomitant disease (E) without acute concomitant disease	Kaplan–Meier curve, log-rank test HR	Significant differences for all comparisons except between A and B	Categorical	–	Inpatient death – B vs A: 3.25 (1.79−5.91) – C vs A: 6.50 (3.43−12.29) 6-week survival – B vs A: 2.81 (1.37−5.77) – C vs A: 9.41 (4.68−18.92) – E vs D: 0.41 (0.22−0.73) 3-week survival – B vs A: 1.50 (0.74−3.01) – C vs A: 5.45 (2.93−10.12) – E vs D: 0.58 (0.36−0.93)	–	Male, presence of acute concomitant diseases, PPI total score
Shatri et al. (2021)	(A) PPI ≤ 4 (B) 4 < PPI ≤ 6 (C) PPI > 6	Kaplan–Meier curve, log-rank test HR	Yes	Categorical	– B vs A: 2.02 (1.11−3.68) – C vs A: 4.22 (2.67−6.66)	–	–	–
Stone et al. (2008)	PPI ≤ 4 4 < PPI ≤ 6 PPI > 6	Median survival and 95% CI HR	Yes	Continuous	1.36 (1.29−1.43)	–	–	–
Subramaniam et al. (2013)	PPI ≤ 4 4 < PPI ≤ 6 PPI > 6	Kaplan–Meier curve, log-rank test HR	Yes	Continuous	1.33 (1.26−1.40)	–	–	–
Trejo-Ayala et al. (2018)	PPI < 4 4 ≤ PPI < 6 PPI ≥ 6	Kaplan–Meier curve, log-rank test RR	No	Categorical	–	–	RR (0 vs > 0) = 3.200 (0.492−20.809)	–

PPI = Palliative Prognostic Index; HR = hazard ratio; RR = risk ratio; OR = odds ratio;, CI = confidence intervals;, BMI = body mass index;, EGFR = estimated glomerular filtration rate; ECOG PS = Eastern Cooperative Oncology Group Performance Status; CRP = C-reactive protein; NLR = neutrophil-to-lymphocyte ratio; mPaP = modified Palliative Prognostic Score.

### Risk of bias assessment

**Figure 2.**
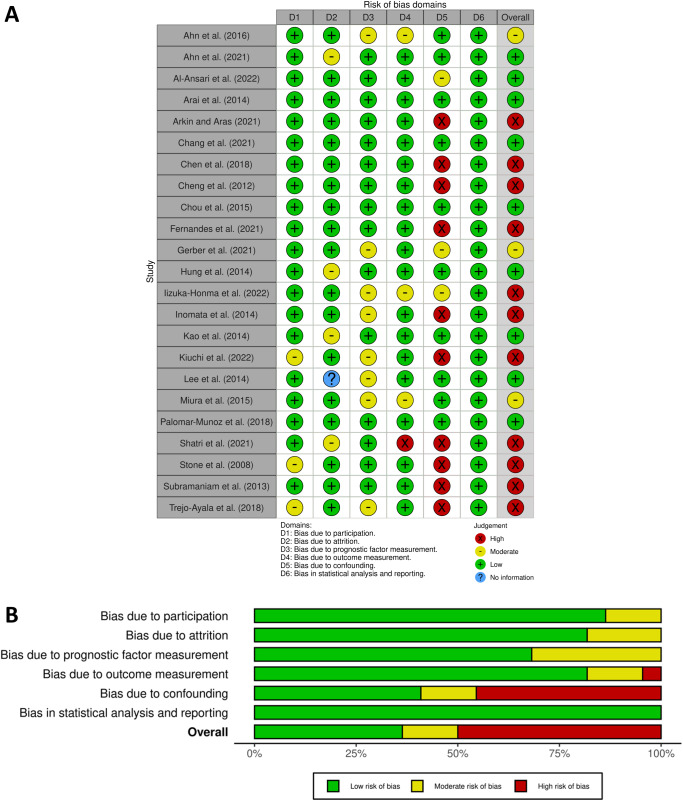
(A) Risk of bias ratings for each study and (B) risk of bias summary graph showing the overall distribution of ratings for each domain.

Figure 2 illustrates the risk of bias ratings. Nine studies were classified as having a low risk of bias (Ahn et al. 2021; Al-Ansari et al. 2022; Arai et al. 2014; Chang et al. 2021; Chou et al. 2015; Hung et al. 2014; Kao et al. 2014; Lee et al. 2014; Palomar-Muñoz et al. 2018), 3 as moderate risk (Ahn et al. 2016; Gerber et al. 2021; Miura et al. 2015), and 11 as high risk (Arkın and Aras 2021; Chen et al. 2018; Cheng et al. 2012; Fernandes et al. 2021; Iizuka-Honma et al. 2023; Inomata et al. 2014; Kiuchi et al. 2022; Shatri et al. 2021; Stone et al. 2008; Subramaniam et al. 2013; Trejo-Ayala et al. 2018).

In the study participation domain, most studies reported population characteristics well, although some did not specify the recruitment period or exclusion criteria. Study attrition was low in most studies, but some only analyzed a subset of participants from larger cohorts, potentially limiting the generalizability of outcomes. In certain studies, those lost to follow-up were excluded, leading to unclear attrition rates. For prognostic factor measurement, the risk of bias was generally low for studies that recorded PPI assessments during the first consultation. However, retrospective studies that calculated scores from available data may have been affected by the quality of clinical documentation. Some studies did not specify who completed the assessments. Outcome measurements were generally well-reported, though a few studies did not specify the duration of follow-up or how the date of death was determined. The risk of bias for study confounding was high or moderate when studies did not adjust for or specify relevant covariates.

### Synthesis results

Detailed GRADE ratings are provided in Table S9. Due to the limited number of studies (less than 10 per meta-analysis), subgroup analyses based on study design, setting, risk of bias, and assessment for publication bias could not be conducted.

### PPI scores as categorical variables

#### PPI > 6 vs PPI ≤ 4 risk groups

**Figure 3.**
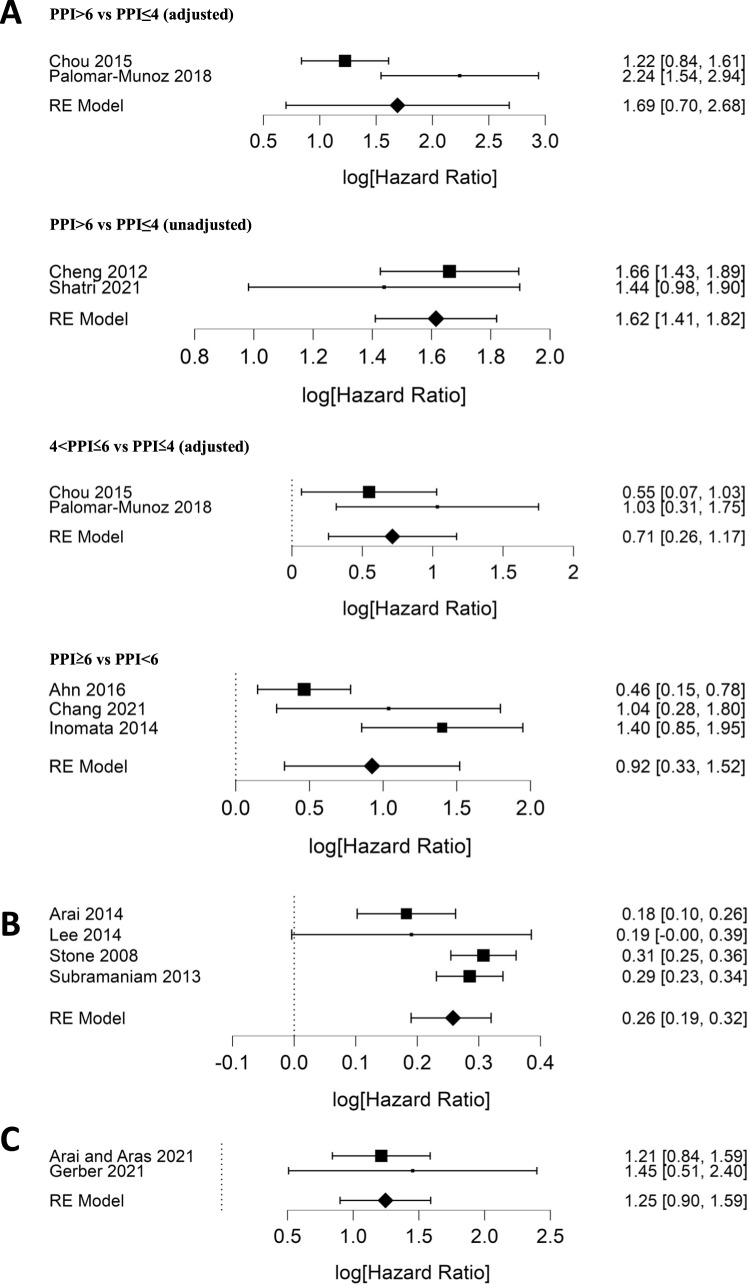
(A) Forest plots for meta-analysis of adjusted and unadjusted hazard ratios for associations between PPI (as a categorical variable) and risk of death for cutoffs 4 and 6. (B) Forest plot for meta-analysis of hazard ratios for the association between PPI (as a continuous variable) and risk of death. (C) Forest plot for meta-analysis of unadjusted risk ratios for inpatient death. Note that the figures show log[hazard ratio] and 95% CI – the results in the main text are in hazard ratio and 95% CI.

The pooled adjusted HR was 5.42 (95% CI 2.01–14.59, *p* = 0.0009) (Chou et al. 2015; Palomar-Muñoz et al. 2018), with considerable heterogeneity (*I*^2^ = 84%, *p* = 0.012) (*n* = 539, high-quality evidence). The pooled unadjusted HR (Cheng et al. 2012; Shatri et al. 2021) was 5.05 (95% CI 4.10–6.17, *p* < 0.00001) with nonsignificant heterogeneity (*I*^2^ = 0%, *p* = 0.40) (*n* = 783, high-quality evidence) (Fig. 3A).

#### 4 < PPI ≤ 6 vs PPI ≤ 4 risk groups

Two studies were pooled (Chou et al. 2015; Palomar-Muñoz et al. 2018), yielding as adjusted HR of 2.04 (95% CI 1.30–3.21, *p* = 0.002) with nonsignificant heterogeneity (*I*^2^ = 17.4%, *p* = 0.271) (*n* = 539, high-quality evidence) (Fig. 3A).

#### PPI ≥ 6 vs PPI < 6 risk groups

Three studies (Ahn et al. 2016; Chang et al. 2021; Inomata et al. 2014) were included in the meta-analysis, with a pooled adjusted HR of 2.52 (95% CI 1.39–4.58, *p* = 0.002), showing considerable heterogeneity (*I*^2^ = 74.5%, *p* = 0.01) (n = 333, moderate quality evidence) (Fig. 3A).

### PPI scores as a continuous variable

Four studies analyzed PPI as continuous variables (Arai et al. 2014; Lee et al. 2014; Stone et al. 2008; Subramaniam et al. 2013). The pooled unadjusted HR was 1.30 (95% CI 1.22–1.38, *p* < 0.00001) with substantial heterogeneity (*I*^2^ = 60%, *p* = 0.06) (*n* = 815, low-quality evidence) (Fig. 3B). This indicates that for each 1-point increase in PPI score, there is a 30% higher risk of mortality.

### Other comparisons

Only 3 studies used the PPI thresholds of 2 and 4 for survival analyses, as defined in the original study (Morita et al. 1999) (Chen et al. 2018; Iizuka-Honma et al. 2023; Kao et al. 2014) (Table 2). Other comparisons that could not be meta-analyzed are presented in Table 2 (Ahn et al. 2021; Cheng et al. 2012; Kiuchi et al. 2022; Miura et al. 2015).

### PPI scores as dichotomous variables (RR or OR)

Six studies reported dichotomous outcomes (Al-Ansari et al. 2022; Arkın and Aras 2021; Fernandes et al. 2021; Gerber et al. 2021; Lee et al. 2022; Trejo-Ayala et al. 2018) (Table 2). Two studies (Arkın and Aras 2021; Gerber et al. 2021) were included in the meta-analysis, yielding a pooled unadjusted RR of 3.48 (95% CI 2.46–4.91, *p* < 0.00001) for PPI ≤ 4 vs PPI > 6 in predicting inpatient death (*n* = 274, high-quality evidence). Heterogeneity was nonsignificant (*I*^2^ = 0%, *p* = 0.64) (Fig. 3C). Findings from other studies that could not be meta-analyzed are presented in Table 2.

### Change in PPI

Three studies investigated changes in PPI scores as a predictor of survival (Arai et al. 2014; Hung et al. 2014; Kao et al. 2014) (Table 2). Hung et al. (2014) and Kao et al. (2014) examined the same patient cohort, but Hung et al. (2014) only involved those with PPI > 6 (poor prognosis). Arai et al. (2014) calculated the change in PPI per day. For Kao et al. (2014), the median survival was the shortest for the group with <0 change in score, followed by the 0 and >0 groups. In Hung et al. (2014), the group with >20% change in score had the shortest median overall survival, followed by the 0–20%, 0, −20 to 0%, and <−20% change groups. Although the studies used different methods to categorize and calculate changes in PPI, most comparisons indicated that changes in PPI score were a statistically significant prognostic factor for survival.

## Discussion

### Main findings

This review is the first to confirm an independent association between PPI scores and survival in advanced cancer patients. It expands on the findings of a previous systematic review, which concluded that higher PPI scores significantly predicted a shorter survival period based solely on unadjusted HR (Liu et al. 2018). Our review includes more recent studies, larger sample sizes, and an analysis of both adjusted and adjustable effect sizes. Additionally, we assessed the risk of bias and the certainty of evidence using the GRADE framework, an evaluation that was not conducted in the previous review (Liu et al. 2018). Building on the findings of the original PPI development study (Morita et al. 1999), all meta-analyses in this review found that the association between PPI and survival remained significant even after adjusting for covariates, with significant differences in survival among the risk groups.

Most included studies conducted an initial patient assessment using the PPI upon admission. However, unexpected events at the end-of-life are common, and reasons for hospitalizations can vary, meaning that the first assessment might not entirely reflect the patient’s overall prognosis. One study found that cancer patients admitted to the palliative care unit with treatable acute conditions (e.g. infections, hemorrhage) were more likely to survive than those admitted due to cancer-related issues (e.g. refractory symptoms, disease progression). Additionally, no significant differences in survival were observed among risk groups when using PPI at discharge (Palomar-Muñoz et al. 2018). This highlights the need for caution when interpreting PPI scores across different patient populations and time points.

Changes in PPI scores could also serve as a significant prognostic factor in predicting survival, particularly in capturing sudden shifts in patients’ conditions during end-of-life care. A study found that worsened symptom scores 1 week after admission were associated with shorter survival compared to patients with improved symptoms. In contrast, those with stable and improved symptom scores showed no significant differences in survival (Suh et al. 2022). Similarly, 3 studies (Arai et al. 2014; Hung et al. 2014; Kao et al. 2014) found significant associations between change in PPI scores and survival outcomes. In addition, Kao et al. (2014) found a model combining the initial PPI score with the change in score had the highest *c*-statistic, further supporting the importance of monitoring PPI score changes over time. The Model combining the initial PPI score with changes in the score proved to be a better predictor of 30-day survival than using the initial score, Week 1 PPI score or score change individually. Another study found that a second PPI assessment conducted on Days 3–5 in hospice residents had better discriminative performance than the first assessment at admission (Subramaniam et al. 2019). The studies (Arai et al. 2014; Hung et al. 2014; Kao et al. 2014) included employed different methods for calculating PPI. Future research should standardize these calculation methods to provide more reliable conclusions regarding the utility of PPI in prognostication.

PPI as a continuous variable may also have prognostic significance. This was observed in another study involving older adults receiving home palliative care (7.5% had cancer), which reported a 1.51-fold increased probability of death for each unit increase in PPI (Moretti et al. 2019). An included study (Gerber et al. 2021) reported an OR of 0.74, suggesting that a lower overall PPI score significantly predicted survival to discharge. However, this result became nonsignificant in multivariate analysis. Hence, the utility of PPI scores as a continuous variable warrants further research.

Finally, we found that the risk of inpatient death was significantly higher for patients with a PPI > 6 compared to those with a PPI ≤ 4. This finding aligns with a previous study, which reported that the mean PPI scores of patients who died in the hospital were significantly higher than those of patients who survived to discharge (8.2 ± 3.8 vs 3.2 ± 2.9, *p* < 0.001) (Alshemmari et al. 2012).

### What this study adds

PPI is not only a reasonably accurate prognostic tool for predicting <3- and <6-week survival in cancer patients (Yoong et al. 2023), but the findings of this review also suggest that a higher PPI score is a strong and independent prognostic factor for poorer survival outcomes in advanced cancer patients. Furthermore, the PPI could support current clinical practice guidelines, which recommend the early integration of palliative care into standard oncology treatment for patients with advanced cancer receiving concurrent active treatment (Corsi et al. 2019; Ferrell et al. 2017; Lee et al. 2022). By assisting clinicians in identifying cancer patients suitable for early palliative care, the PPI could enhance clinical decision-making, helping clinicians determine whether additional curative treatment may benefit the patient or if palliative care should be initiated (Cohen and Miner 2019; Hasegawa et al. 2015; Pobar et al. 2021).

PPI could also be valuable when an objective estimate of survival is needed, e.g. determining participants’ eligibility for clinical trials (Chu et al. 2020; Simms et al. 2013), conducting risk stratification in stratified randomized trials, or avoiding bias in treatment effect estimation by adjusting for PPI (Halabi and Owzar 2010). It may also help identify patients with poorer outcomes, thereby encouraging clinical trial participation for novel or experimental treatments (Gospodarowicz et al. 2001). A study examining the impact of palliative radiotherapy on gastric cancer patients’ symptoms found that, after adjusting for baseline PPI (since patients with limited life expectancy often experience worsening symptoms), shortness of breath, pain, and distress significantly improved over 8 weeks. Additionally, higher PPI scores were associated with higher symptom scores at all time points (Kawamoto et al. 2022). Another study identified a baseline PPI of >2 as a reliable predictor of death within 2 months in patients with advanced gastric cancer patients, suggesting it may be suitable for guiding single-fraction radiotherapy (Sekii et al. 2023).

Although various prognostic factors and prediction models have been identified for cancer patients, many were specific to certain cancer types or complications, limiting their clinical applicability to the broader cancer population (Owusuaa et al. 2022). A prediction model that is simple to use, applicable to heterogeneous cancer populations, and accessible to medical specialists, general practitioners, and nurses is highly desirable, as it could aid in treatment planning and advance care decisions (Owusuaa et al. 2022). Some studies have pointed out the challenges of using certain prognostic tools due to the unavailability of blood test results (Baba et al. 2015; Kishino et al. 2022). In addition, many existing prediction models lack external validation, and model calibration is rarely assessed, underscoring the need for well-performing, validated models that are applicable to most cancer patients (Kreuzberger et al. 2020; Owusuaa et al. 2022). The PPI tool could help address this gap, as it has been widely validated and accepted across diverse settings and cancer populations.

### Strengths and limitations of the study

This is the first meta-analysis to report an independent association between PPI scores and survival, and it represents the most comprehensive systematic review on the prognostic utility of PPI to date. The finding may offer valuable insights that can benefit both clinicians and researchers.

This review has several limitations. First, only articles in English were included, which may have resulted in the exclusion of relevant studies published in other languages. There were also limited studies in each meta-analysis, so the results should be interpreted with caution. As a result, subgroup analysis and tests for publication bias could not be conducted. We also did not estimate HR from the published Kaplan–Meier curves, as most studies did not report numbers at risk, which hindered this estimation. Moreover, studies that did not provide effect sizes (e.g. only reporting a significant log-rank test) were excluded, meaning this review does not represent all available literature on the association between PPI and survival. Despite these limitations, this review aimed to evaluate whether PPI is a prognostic factor for survival; thus, making the synthesis of time-to-event outcome measures the most appropriate approach.

### Implications for research and practice

The PPI was initially developed using a heterogeneous sample of patients with different types of cancers (Morita et al. 1999). Subsequently, its utility has been investigated and validated in specific cancer types, including lung cancer (Arkın and Aras 2021; Inomata et al. 2014), hematological malignancies (Chang et al. 2021; Chou et al. 2015; Iizuka-Honma et al. 2023; Trejo-Ayala et al. 2018), and ovarian cancer (Kiuchi et al. 2022). One study also found that PPI was associated with survival in patients with non-Hodgkin’s lymphoma but not in those with acute myeloid leukemia in the palliative care setting (Yamane et al. 2023). Future research should continue to explore whether the prognostic utility of PPI differs across cancer types, patient care settings (such as acute wards, home palliative care, hospices, etc.) and stages of the cancer treatment journey (e.g. during active treatment or palliative care), similar to how the Glasgow Prognostic Score has been comprehensively evaluated for various cancers (He et al. 2018; Tong et al. 2020; Wu et al. 2021).

Most of the included studies had a moderate to high risk of bias, highlighting the need for improving reporting in future research. To strengthen credibility and ensure the findings are more reliable for practical application, future studies should adhere to established reporting guidelines (Altman et al. 2012; Hayden et al. 2013). It is also crucial to report adjusted prognostic effect measures, as these are important for quantifying the extent of the increased mortality risk across PPI risk groups. We observed that the categorization of PPI risk groups was inconsistent, with only 3 out of 23 studies using the risk groups defined in the original development study, and a maximum of 3 studies testing the same comparison. As a result, our meta-analyses were limited by the small number of studies. Further research should validate our findings by further examining the predictive value of PPI score categories (PPI ≤ 2, 2 < PPI ≤ 4, and PPI > 4) as defined in the original development study.

## Conclusion

Higher PPI scores were strongly associated with poorer survival outcomes in advanced cancer patients. While the limited number of studies in each risk group comparison constrained our meta-analyses, the findings were consistent in both direction and significance. Future studies should adhere to the risk categories defined in the original development study and report adjusted effect estimates with 95% CI to strengthen the evidence base.

## Supporting information

Yoong et al. supplementary material Yoong et al. supplementary material

## Supplementary material

The supplementary material for this article can be found at https://doi.org/.10.1017/S1478951525000021.
