# Supplementary material for: Prognostic utility of Palliative Prognostic Index in advanced cancer: A systematic review and meta-analysis
Source: Palliat Support Care. 2025 Jan 21;23:e80. doi: 10.1017/S1478951525000021 (PMC13166367; doi:10.1017/S1478951525000021)
Supplement: Yoong et al. supplementary material [file S1478951525000021sup001.docx]

**Supplementary File**

**Table S1. PRISMA checklist**

| **Section and Topic** | **Item #** | **Checklist item** | **Location where item is reported** |
| --- | --- | --- | --- |
| **TITLE** | | |  |
| Title | 1 | Identify the report as a systematic review. | Page 1 |
| **ABSTRACT** | | |  |
| Abstract | 2 | See the PRISMA 2020 for Abstracts checklist. | Page 1-2 |
| **INTRODUCTION** | | |  |
| Rationale | 3 | Describe the rationale for the review in the context of existing knowledge. | Page 4 |
| Objectives | 4 | Provide an explicit statement of the objective(s) or question(s) the review addresses. | Page 4 |
| **METHODS** | | |  |
| Eligibility criteria | 5 | Specify the inclusion and exclusion criteria for the review and how studies were grouped for the syntheses. | Page 5 |
| Information sources | 6 | Specify all databases, registers, websites, organisations, reference lists and other sources searched or consulted to identify studies. Specify the date when each source was last searched or consulted. | Page 5 |
| Search strategy | 7 | Present the full search strategies for all databases, registers and websites, including any filters and limits used. | Supplementary Methods |
| Selection process | 8 | Specify the methods used to decide whether a study met the inclusion criteria of the review, including how many reviewers screened each record and each report retrieved, whether they worked independently, and if applicable, details of automation tools used in the process. | Page 5 |
| Data collection process | 9 | Specify the methods used to collect data from reports, including how many reviewers collected data from each report, whether they worked independently, any processes for obtaining or confirming data from study investigators, and if applicable, details of automation tools used in the process. | Page 6 |
| Data items | 10a | List and define all outcomes for which data were sought. Specify whether all results that were compatible with each outcome domain in each study were sought (e.g. for all measures, time points, analyses), and if not, the methods used to decide which results to collect. | Page 6 |
|  | 10b | List and define all other variables for which data were sought (e.g. participant and intervention characteristics, funding sources). Describe any assumptions made about any missing or unclear information. | Page 6 |
| Study risk of bias assessment | 11 | Specify the methods used to assess risk of bias in the included studies, including details of the tool(s) used, how many reviewers assessed each study and whether they worked independently, and if applicable, details of automation tools used in the process. | Page 6 |
| Effect measures | 12 | Specify for each outcome the effect measure(s) (e.g. risk ratio, mean difference) used in the synthesis or presentation of results. | Page 6 |
| Synthesis methods | 13a | Describe the processes used to decide which studies were eligible for each synthesis (e.g. tabulating the study intervention characteristics and comparing against the planned groups for each synthesis (item #5)). | Page 7 |
|  | 13b | Describe any methods required to prepare the data for presentation or synthesis, such as handling of missing summary statistics, or data conversions. | Page 7 |
|  | 13c | Describe any methods used to tabulate or visually display results of individual studies and syntheses. | Page 7 |
|  | 13d | Describe any methods used to synthesize results and provide a rationale for the choice(s). If meta-analysis was performed, describe the model(s), method(s) to identify the presence and extent of statistical heterogeneity, and software package(s) used. | Page 7 |
|  | 13e | Describe any methods used to explore possible causes of heterogeneity among study results (e.g. subgroup analysis, meta-regression). | Page 8 |
|  | 13f | Describe any sensitivity analyses conducted to assess robustness of the synthesized results. | N/A |
| Reporting bias assessment | 14 | Describe any methods used to assess risk of bias due to missing results in a synthesis (arising from reporting biases). | N/A |
| Certainty assessment | 15 | Describe any methods used to assess certainty (or confidence) in the body of evidence for an outcome. | Page 7 |
| **RESULTS** | | |  |
| Study selection | 16a | Describe the results of the search and selection process, from the number of records identified in the search to the number of studies included in the review, ideally using a flow diagram. | Page 8 and Figure 1 |
|  | 16b | Cite studies that might appear to meet the inclusion criteria, but which were excluded, and explain why they were excluded. | Supplementary methods |
| Study characteristics | 17 | Cite each included study and present its characteristics. | Page 8 and Table 1 |
| Risk of bias in studies | 18 | Present assessments of risk of bias for each included study. | Page 9 and Figure 2 |
| Results of individual studies | 19 | For all outcomes, present, for each study: (a) summary statistics for each group (where appropriate) and (b) an effect estimate and its precision (e.g. confidence/credible interval), ideally using structured tables or plots. | Table 2 |
| Results of syntheses | 20a | For each synthesis, briefly summarise the characteristics and risk of bias among contributing studies. | N/A |
|  | 20b | Present results of all statistical syntheses conducted. If meta-analysis was done, present for each the summary estimate and its precision (e.g. confidence/credible interval) and measures of statistical heterogeneity. If comparing groups, describe the direction of the effect. | Page 10-12 |
|  | 20c | Present results of all investigations of possible causes of heterogeneity among study results. | N/A |
|  | 20d | Present results of all sensitivity analyses conducted to assess the robustness of the synthesized results. | N/A |
| Reporting biases | 21 | Present assessments of risk of bias due to missing results (arising from reporting biases) for each synthesis assessed. | N/A |
| Certainty of evidence | 22 | Present assessments of certainty (or confidence) in the body of evidence for each outcome assessed. | Page 10-12 and Supplementary Results |
| **DISCUSSION** | | |  |
| Discussion | 23a | Provide a general interpretation of the results in the context of other evidence. | Page 12-17 |
|  | 23b | Discuss any limitations of the evidence included in the review. | Page 16 |
|  | 23c | Discuss any limitations of the review processes used. | Page 16 |
|  | 23d | Discuss implications of the results for practice, policy, and future research. | Page 16-17 |
| **OTHER INFORMATION** | | |  |
| Registration and protocol | 24a | Provide registration information for the review, including register name and registration number, or state that the review was not registered. | Page 5 |
|  | 24b | Indicate where the review protocol can be accessed, or state that a protocol was not prepared. | Page 5 |
|  | 24c | Describe and explain any amendments to information provided at registration or in the protocol. | None |
| Support | 25 | Describe sources of financial or non-financial support for the review, and the role of the funders or sponsors in the review. | N/A |
| Competing interests | 26 | Declare any competing interests of review authors. | N/A |
| Availability of data, code and other materials | 27 | Report which of the following are publicly available and where they can be found: template data collection forms; data extracted from included studies; data used for all analyses; analytic code; any other materials used in the review. | The review itself and online supplementary material |

*From:*  Page MJ, McKenzie JE, Bossuyt PM, Boutron I, Hoffmann TC, Mulrow CD, et al. The PRISMA 2020 statement: an updated guideline for reporting systematic reviews. BMJ 2021;372:n71. doi: 10.1136/bmj.n71

For more information, visit: <http://www.prisma-statement.org/>

**Supplementary methods. Search strategy and reasons for exclusion**

Date of search: 16 February 2024

**Table S2. PubMed**

| **Search number** | **Query** | **Results** |
| --- | --- | --- |
| #1 | "terminal care"[Title/Abstract] OR "hospice care"[Title/Abstract] OR "end of life"[Title/Abstract] OR "terminally ill"[Title/Abstract] OR "advance care"[Title/Abstract] OR "palliative care"[Title/Abstract] OR "terminal care"[MeSH Terms] OR "hospice care"[MeSH Terms] OR "terminally ill"[MeSH Terms] OR "palliative care"[MeSH Terms] OR "neoplasms"[MeSH Terms] OR "cancer"[Title/Abstract] | 4,661,561 |
| #2 | Palliative prognostic index [Title/Abstract] | 118 |
| #3 | Prognos*[Title/Abstract] OR predict*[Title/Abstract] OR survival[Title/Abstract] OR mortality[Title/Abstract] | 4,247,538 |
| #4 | #1 and #2 and #3 | 117 |

**Table S3. Embase**

| **Search number** | **Query** | **Results** |
| --- | --- | --- |
| #1 | 'palliative therapy':ti,ab,kw OR 'terminal disease':ti,ab,kw OR 'hospice care':ti,ab,kw OR 'malignant neoplasm':ti,ab,kw OR 'terminal care':ti,ab,kw | 24,292 |
| #2 | 'palliative prognostic index':ti,ab,kw | 177 |
| #3 | ‘Prognosis’:ti,ab,kw OR ‘predict’:ti,ab,kw OR ‘survival’:ti,ab,kw OR ‘mortality’:ti,ab,kw | 4,079,188 |
| #4 | #1 and #2 and #3 | 15 |

**Table S4. ScienceDirect** (limit to English)

| **Query** | **Results** |
| --- | --- |
| (Palliative care OR terminally ill OR terminal care OR advanced cancer OR hospice care OR cancer) AND “palliative prognostic index” | 143 |

**Table S5. Web of Science** (limit to articles, English)

| **Search number** | **Query** | **Results** |
| --- | --- | --- |
| #1 | Topic = (Palliative care OR terminally ill OR terminal care OR advance care OR hospice care OR cancer) | 595 |
| #2 | Topic = “palliative prognostic index” |  |
| #3 | Topic = Prognos* OR predict* OR survival OR mortality |  |
| #4 | #1 and #2 and #3 |  |

**Table S6. CINAHL** (limit: English)

| **Search number** | **Query** | **Results** |
| --- | --- | --- |
| #1 | palliative care or end of life care or terminal care or hospice care or cancer | 68 |
| #2 | “palliative prognostic index” |  |
| #3 | Prognos* OR predict* OR survival OR mortality |  |
| #4 | #1 and #2 and #3 |  |

**Table S7. ProQuest Dissertations & Theses Global** via Web of Science (limit to English)

| **Search number** | **Query** | **Results** |
| --- | --- | --- |
| #1 | Topic = (Palliative care OR terminally ill OR terminal care OR advance care OR hospice care OR cancer) | 6 |
| #2 | Topic = “palliative prognostic index” |  |
| #3 | Topic = Prognos* OR predict* OR survival OR mortality |  |
| #4 | #1 and #2 and #3 |  |

**Table S8. Reasons for exclusion**

| **Number** | **Title** | **Year** | **Authors** | **doi** | **Notes** |
| --- | --- | --- | --- | --- | --- |
| 1 | Utility of Palliative Prognostic Index in Predicting Survival Outcomes in Patients With Hematological Malignancies in the Acute Ward Setting. | 2022 | Lee, Shu-Hui and Chou, Wen-Chi and Yang, Hsin-Yi and Chen, Chia-Chia and Chang, Hung and Wang, Po-Nan and Kuo, Ming-Chung and Kao, Yu-Feng and Ho, Lun-Hui and Hsueh, Shun-Wen and Kao, Chen-Yi and Hsueh, William Harrison and Hung, Chia-Yen and Hung, Yu-Shin | 10.1177/10499091211028820 | Unclear if advanced cancer |
| 2 | Prospective Comparison of Prognostic Scores in Palliative Care Cancer Populations. | 2012 | Maltoni, Marco and Scarpi, Emanuela and Pittureri, Cristina and Martini, Francesca and Montanari, Luigi and Amaducci, Elena and Derni, Stefania and Fabbri, Laura and Rosati, Marta and Amadori, Dino and Nanni, Oriana | 10.1634/theoncologist.2011-0397 | Effect size not hazard, risk or odds ratios |
| 3 | Utility of Prognostic Prediction Models in the Terminal Stage of Gastrointestinal Cancer. | 2020 | Kadokura M and Okuwaki T and Imagawa N and Shimamura N and Takada H and Amemiya F | 10.1007/s12029-019-00270-5 | Effect size not hazard, risk or odds ratios |
| 4 | Prognostic model for patients with advanced cancer using a combination of routine blood test values. | 2021 | Miyagi T and Miyata S and Tagami K and Hiratsuka Y and Sato M and Takeda I and Kohata K and Satake N and Shimokawa H and Inoue A | 10.1007/s00520-020-05937-5 | Effect size not hazard, risk or odds ratios |
| 5 | Effects of Antibiotics on Respiratory Symptoms in Terminally Ill Cancer Patients With Pneumonia: A Multicenter Cohort Study. | 2022 | Odagiri T and Maeda I and Masanori Mori and Morita T and Kaneishi K and Junko Nozato and Kazuhiro Kosugi and Higashibata T and Hamano J and Shimoinaba J and Nishi T and Kawashima N | 10.1177/10499091211058156 | Effect size not related to patient survival |
| 6 | Prognostic evaluation in palliative care: final results from a prospective cohort study. | 2019 | Ermacora P and Mazzer M and Isola M and Pascoletti G and Gregoraci G and Basile D and De Carlo E and Merlo V and Luz O and Cattaruzza M and Orlando A and Bozza C and Pella N and Sacco CS and Puglisi F and Fasola G and Aprile G | 10.1007/s00520-018-4463-z | Effect size not hazard, risk or odds ratios |
| 7 | Inpatient Hospice Palliative Care Unit and Palliative Consultation Service Enhance Comprehensive Quality of Life Outcomes in Terminally Ill Cancer Patients: A Prospective Longitudinal Study. | 2021 | Chang LF and Wu LF and Lin CK and Ho CL and Hung YC and Pan HH | 10.3390/ijerph18178992 | Effect size not related to patient survival |
| 8 | Are Prognostic Scores Better Than Clinician Judgment? A Prospective Study Using Three Models. | 2022 | Hiratsuka Y and Suh SY and Hui D and Morita T and Mori M and Oyamada S and Amano K and Imai K and Baba M and Kohara H and Hisanaga T and Maeda I and Hamano J and Inoue A | 10.1016/j.jpainsymman.2022.06.008 | Effect size not hazard, risk or odds ratios |
| 9 | Experience of symptom control, anxiety and associating factors in a palliative care unit evaluated with Support Team Assessment Schedule Japanese version. | 2021 | Ito T and Tomizawa E and Yano Y and Takei K and Takahashi N and Shaku F | 10.1038/s41598-021-97143-4 | Effect size not related to patient survival |
| 10 | Value of Traditional Chinese Medicine syndrome differentiation in predicting the survival time of patients with advanced cancer. | 2021 | Gu XL and Chen ML and Liu MH and Zhang Z and Zhao WW and Cheng WW | 10.19852/j.cnki.jtcm.20210310.001 | Effect size not hazard, risk or odds ratios |
| 11 | Comparison of Symptom Severity and Progression in Advanced Cancer Patients Among Different Care Settings: A Secondary Analysis. | 2023 | Shiraishi R and Kizawa Y and Mori M and Maeda I and Hatano Y and Ishiki H and Miura T and Yokomichi N and Kodama M and Inoue K and Otomo S and Yamaguchi T and Hamano J | 10.1089/pmr.2023.0011 | Effect size not related to patient survival |
| 12 | A Comparison of the Accuracy of Clinician Prediction of Survival Versus the Palliative Prognostic Index. | 2018 | Farinholt P and Park M and Guo Y and Bruera E and Hui D | 10.1016/j.jpainsymman.2017.11.028 | Effect size not hazard, risk or odds ratios |
| 13 | Clinical Features of Patients With Hematological Malignancies Treated at the Palliative Care Unit. | 2023 | Yamane H and Ochi N and Mimura A and Kosaka Y and Ichiyama N and Kawahara T and Nagasaki Y and Nakanishi H and Takigawa N | 10.1089/pmr.2023.0028 | Effect size not hazard, risk or odds ratios |
| 14 | The Magnitude and Effects of Early Integration of Palliative Care Into Oncology Service Among Adult Advanced Cancer Patients at a Tertiary Care Hospital. | 2021 | Ghabashi EH and Sharaf BM and Kalaktawi WA and Calacattawi R and Calacattawi AW | 10.7759/cureus.15313 | Effect size not hazard, risk or odds ratios |
| 15 | Validation of the Palliative Prognostic Index, Performance Status-Based Palliative Prognostic Index and Chinese Prognostic Scale in a home palliative care setting for patients with advanced cancer in China. | 2020 | Zhou J and Xu S and Cao Z and Tang J and Fang X and Qin L and Zhou F and He Y and Zhong X and Hu M and Wang Y and Lu F and Bao Y and Dai X and Wu Q | 10.1186/s12904-020-00676-0 | Effect size not hazard, risk or odds ratios |
| 16 | Symptoms and Prognoses of Patients With Breast Cancer and Malignant Wounds in Palliative Care Units: The Multicenter, Prospective, Observational EASED Study. | 2023 | Takeda Y and Ishiki H and Oyamada S and Otani H and Maeda I and Yamaguchi T and Hamano J and Mori M and Morita T | 10.1177/10499091231219855 | Effect size not hazard, risk or odds ratios |
| 17 | Impact of a Six-Year Project to Enhance the Awareness of Community-Based Palliative Care on the Place of Death. | 2018 | Murakami N and Tanabe K and Morita T and Fujikawa Y and Koseki S and Kajiura S and Nakajima K and Hayashi R | 10.1089/jpm.2017.0696 | Effect size not related to patient survival |
| 18 | Discharge to home from a palliative care unit: impact on survival and factors associated with home death after the discharge: a cohort study. | 2023 | Murakami N and Kajiura S and Tanabe K and Tsukada K and Shibata K and Minabe Y and Morita T and Hayashi R | 10.1186/s12904-023-01314-1 | Effect size not related to patient survival |
| 19 | The determinants of patients in a palliative care unit being discharged home in Japan. | 2014 | Amano K and Nishiuchi Y and Baba M and Kawasaki M and Nakajima S and Wakayama H and Watakabe A and Kunimoto H and Morita T | 10.1177/1049909113484384 | Effect size not related to patient survival |
| 20 | Predictors of 3-month mortality with muscle ultrasound and palliative prognostic tools among patients admitted to palliative care units. | 2023 | Kaya ZI and Öztürk Y and Gürcü S and Uncu G and Uçan A and Eşme M and Kaya BY and Balci C | 10.3904/kjim.2022.308 | Mixed sample of various diagnoses |
| 21 | Prognosis prediction with two calculations of Palliative Prognostic Index: further prospective validation in hospice cancer patients with multicentre study. | 2019 | Subramaniam S and Dand P and Ridout M and Cawley D and Miller S and Valli P and Bright R and O'Neill B and Wilcocks T and Parker G and Harris D | 10.1136/bmjspcare-2017-001418 | Effect size not hazard, risk or odds ratios |
| 22 | Phase angle for prognostication of survival in patients with advanced cancer: preliminary findings. | 2014 | Hui D and Bansal S and Morgado M and Dev R and Chisholm G and Bruera E | 10.1002/cncr.28624 | Effect size not hazard, risk or odds ratios |
| 23 | The Palliative Prognostic Index: a scoring system for survival prediction of terminally ill cancer patients. | 1999 | Morita T and Tsunoda J and Inoue S and Chihara S | 10.1007/s005200050242 | Effect size not hazard, risk or odds ratios |
| 24 | The palliative prognostic index for the prediction of survival and in-hospital mortality of patients with advanced cancer in Kuwait. | 2012 | Alshemmari S and Ezzat H and Samir Z and Refaat S and Alsirafy SA | 10.1089/jpm.2011.0253 | Effect size not hazard, risk or odds ratios |
| 25 | Who should receive single-fraction palliative radiotherapy for gastric cancer bleeding?: An exploratory analysis of a multicenter prospective observational study (JROSG 17-3). | 2023 | Sekii S and Saito T and Kosugi T and Nakamura N and Wada H and Tonari A and Ogawa H and Mitsuhashi N and Yamada K and Takahashi T and Ito K and Kawamoto T and Araki N and Nozaki M and Heianna J and Murotani K and Hirano Y and Satoh A and Onoe T and Shikama N | 10.1016/j.ctro.2023.100657 | Effect size not hazard, risk or odds ratios |
| 26 | The survival time of terminal cancer patients: prediction based on clinical parameters and simple prognostic scores. | 2014 | Kim AS and Youn CH and Ko HJ and Kim HM | https://doi.org/10.1177/082585971403000104 | Effect size not hazard, risk or odds ratios |
| 27 | Efficacy of palliative radiotherapy for gastric bleeding in patients with unresectable advanced gastric cancer: a retrospective cohort study. | 2015 | Kondoh C and Shitara K and Nomura M and Takahari D and Ura T and Tachibana H and Tomita N and Kodaira T and Muro K | 10.1186/s12904-015-0034-y | Effect size not related to patient survival |
| 28 | Improved accuracy of physicians' survival prediction for terminally ill cancer patients using the Palliative Prognostic Index. | 2001 | Morita T and Tsunoda J and Inoue S and Chihara S | 10.1191/026921601680419474 | Effect size not hazard, risk or odds ratios |
| 29 | Utility of palliative prognostic index and neutrophil-to-lymphocyte ratio in predicting prognosis of end-stage squamous cell carcinoma of head and neck. | 2024 | Higashino M and Sugimoto K and Onishi S and Okabe K and Yasuda C and Tadokoro H and Kawata R | 10.1002/hed.27549 | Effect size not hazard, risk or odds ratios |
| 30 | Use of the palliative prognostic index in a palliative care consultation service in Melbourne, Australia. | 2010 | Yoong J and Atkin N and Le B | 10.1016/j.jpainsymman.2009.08.001 | Effect size not hazard, risk or odds ratios |
| 31 | Liver-specific metastases as an independent prognostic factor in cancer patients receiving hospice care in hospital | 2023 | Huang, K.-S. and Huang, Y.-H. and Chen, C.-T. and Chou, C.-P. and Pan, B.-L. and Lee, C.-H. | 10.1186/s12904-023-01180-x | Effect size not hazard, risk or odds ratios |
| 32 | Prognostic factors in patients in the terminal phase of hematological malignancies who received home medical care | 2022 | Miyashita, N. and Onozawa, M. and Fujita, M. and Hosoda, T. and Kawasaki, Y. and Takimoto, M. and Okina, S. and Ohashi, K. | 10.1097/01.HS9.0000852292.38263.b8 | Effect size not hazard, risk or odds ratios |
| 33 | Comparison of the accuracy of clinical prediction of survival and palliative prognostic index for patients with head and neck squamous cell carcinoma in the end-of-life setting | 2022 | Kishino, T. and Monden, N. and Akisada, N. and Hayashi, Y. and Nakamura, M. and Hashimoto, K. and Miyashita, T. and Mori, T. and Hoshikawa, H. | 10.1016/j.anl.2021.06.003 | Effect size not hazard, risk or odds ratios |
| 34 | C-Reactive Protein/Albumin Ratio Is an Independent Prognostic Predictor of Survival in Advanced Cancer Patients Receiving Palliative Care | 2019 | Zhang, J. and Zhang, C. and Li, Q. and Zhang, J. and Gu, X. and Zhao, W. and Chen, M. and Liu, M. and Zhang, Z. and Liao, X. and Cheng, W. | 10.1089/jpm.2019.0102 | Sample includes pediatric patients |
| 35 | Prognosis palliative care study, palliative prognostic index, palliative prognostic score and objective prognostic score in advanced cancer: a prospective comparison | 2021 | Lee, SH and Lee, JG and Choi, YJ and Seol, YM and Kim, H and Kim, YJ and Yi, YH and Tak, YJ and Kim, GL and Ra, YJ and Lee, SY and Cho, YH and Park, EJ and Lee, Y and Choi, J and Lee, SR and Kwon, RJ and Son, SM | 10.1136/bmjspcare-2021-003077 | Effect size not hazard, risk or odds ratios |
| 36 | Validation of the Palliative Prognostic Index and Palliative Prognostic Score in a Palliative Care Consultation Team Setting for Patients With Advanced Cancers in an Acute Care Hospital in Japan | 2014 | Sonoda, H and Yamaguchi, T and Matsumoto, M and Hisahara, K | 10.1177/1049909113506034 | Effect size not hazard, risk or odds ratios |
| 37 | Evaluation and comparison of two prognostic scores and the physicians' estimate of survival in terminally ill patients | 2010 | Stiel, S and Bertram, L and Neuhaus, S and Nauck, F and Ostgathe, C and Elsner, F and Radbruch, L | 10.1007/s00520-009-0628-0 | Effect size not hazard, risk or odds ratios |
| 38 | Predicting survival in patients with advanced cancer in the last weeks of life: How accurate are prognostic models compared to clinicians' estimates? | 2020 | Hui, D and Ross, J and Park, M and Dev, R and Vidal, M and Liu, DN and Paiva, CE and Bruera, E | 10.1177/0269216319873261 | Effect size not hazard, risk or odds ratios |
| 39 | The Utility of Glasgow Prognostic Score and Palliative Prognostic Index in Patients With Head and Neck Squamous Cell Carcinoma Under Palliative Care | 2023 | Kishino, T and Mori, T and Miyashita, T and Ouchi, Y and Samukawa, Y and Fukumura, T and Takahashi, S and Monden, N and Akisada, N and Hayashi, Y and Nakamura, M and Hoshikawa, H | 10.1177/01455613211005114 | Effect size not hazard, risk or odds ratios |
| 40 | A prospective, multicenter cohort study to validate a simple performance status-based survival prediction system for oncologists | 2017 | Yamada, T and Morita, T and Maeda, I and Inoue, S and Ikenaga, M and Matsumoto, Y and Baba, M and Sekine, R and Yamaguchi, T and Hirohashi, T and Tajima, T and Tatara, R and Watanabe, H and Otani, H and Takigawa, C and Matsuda, Y and Ono, S and Ozawa, T and Yamamoto, R and Shishido, H and Yamamoto, N | 10.1002/cncr.30484 | Effect size not hazard, risk or odds ratios |
| 41 | Validation of 2 Prognostic Models in Hospitalized Patients With Advanced Hematological Malignancies in Japan | 2017 | Ohno, E and Abe, M and Sasaki, H and Okuhiro, K | 10.1177/1049909115615567 | Effect size not hazard, risk or odds ratios |
| 42 | Usefulness of Palliative Prognostic Index for Patient With Advanced Cancer in Home Care Setting | 2013 | Hamano, J and Maeno, T and Kizawa, Y and Shima, Y and Maeno, T | 10.1177/1049909112448923 | Effect size not hazard, risk or odds ratios |
| 43 | Prospective Clarification of the Utility of the Palliative Prognostic Index for Patients With Advanced Cancer in the Home Care Setting | 2014 | Hamano, J and Kizawa, Y and Maeno, T and Nagaoka, H and Shima, Y and Maeno, T | 10.1177/1049909113504982 | Effect size not hazard, risk or odds ratios |
| 44 | Predicting prognosis in patients with advanced cancer: A prospective study | 2018 | Tavares, T and Oliveira, M and Gonçalves, J and Trocado, V and Perpétuo, J and Azevedo, A and Machado, F and Barreto, V and Rocha, C | 10.1177/0269216317705788 | Effect size not hazard, risk or odds ratios |
| 45 | Survival prediction for advanced cancer patients in the real world: A comparison of the Palliative Prognostic Score, Delirium-Palliative Prognostic Score, Palliative Prognostic Index and modified Prognosis in Palliative Care Study predictor model | 2015 | Baba, M and Maeda, I and Morita, T and Inoue, S and Ikenaga, M and Matsumoto, Y and Sekine, R and Yamaguchi, T and Hirohashi, T and Tajima, T and Tatara, R and Watanabe, H and Otani, H and Takigawa, C and Matsuda, Y and Nagaoka, H and Mori, M and Tei, Y and Hiramoto, S and Suga, A and Kinoshita, H | 10.1016/j.ejca.2015.04.025 | Effect size not hazard, risk or odds ratios |
| 46 | Delirium Frequency and Risk Factors Among Patients With Cancer in Palliative Care Unit | 2017 | Senel, G and Uysal, N and Oguz, G and Kaya, M and Kadioullari, N and Koçak, N and Karaca, S | 10.1177/1049909115624703 | Effect size not related to patient survival |
| 47 | Comparison of Accuracy Among Prognostic Scores for Predicting Life Expectancy in Korean Patients With Cancer With Weeks of Survival | 2014 | Yoon, SJ and Jung, JG and Kim, JS and Kim, SS and Kim, S | 10.1177/1049909113503486 | Effect size not hazard, risk or odds ratios |
| 48 | Accuracy and usefulness of the Palliative Prognostic Index in a community setting | 2015 | Belanger, E and Tetrault, D and Tradounsky, G and Towers, A and Marchessault, J | 10.12968/ijpn.2015.21.12.602 | Effect size not hazard, risk or odds ratios |
| 49 | Prognostic Models Associated with 6-Month Survival of Patients Admitted to Nursing Homes | 2019 | Esteban-Burgos, AA and El Mansouri-Yachou, J and Muñoz-Ramirez, R and Hueso-Montoro, C and Garcia-Caro, MP and Montoya-Juarez, R | 10.1159/000490243 | Mixed sample of various diagnoses |
| 50 | Predictors of response to corticosteroids for dyspnea in advanced cancer patients: a preliminary multicenter prospective observational study | 2017 | Mori, M and Shirado, AN and Morita, T and Okamoto, K and Matsuda, Y and Matsumoto, Y and Yamada, H and Sakurai, H and Aruga, E and Kaneishi, K and Watanabe, H and Yamaguchi, T and Odagiri, T and Hiramoto, S and Kohara, H and Matsuo, N and Katayama, H and Nishi, T and Matsui, T and Iwase, S | 10.1007/s00520-016-3507-5 | Effect size not related to patient survival |
| 51 | Impact of the Macmillan specialist Care at Home service: a mixed methods evaluation across six sites | 2018 | Johnston, B and Patterson, A and Bird, L and Wilson, E and Almack, K and Mathews, G and Seymour, J | 10.1186/s12904-018-0281-9 | Effect size not hazard, risk or odds ratios |

**Table S9. Detailed GRADE ratings**

**Question: Is PPI score significantly associated with survival in cancer patients?**

| **№ of studies** | **Certainty assessment** | | | | | | **Effect** | | **Certainty** |
| --- | --- | --- | --- | --- | --- | --- | --- | --- | --- |
|  | **Phase of investigation** | **Risk of bias** | **Inconsistency** | **Indirectness** | **Imprecision** | **Other considerations** | **№ of individuals** | **Effect size** **(95% CI)** |  |
| **PPI>6 vs PPI≤4 (adjusted) (assessed with: PPI)** | | | | | | | | | |
| 2 | Phase 3 explanatory studies | not serious | serious^e^ | not serious | not serious | very strong association^d^, dose response gradient | 539 | HR = 5.42 (2.01-14.59) | ⨁⨁⨁⨁ High |
| **PPI>6 vs PPI≤4 (unadjusted) (assessed with: PPI)** | | | | | | | | | |
| 2 | Phase 3 explanatory studies | very serious^b^ | not serious | not serious | not serious | very strong association^d^, dose response gradient | 783 | HR = 5.05 (4.10-6.17) | ⨁⨁⨁⨁ High |
| **4<PPI≤6 vs PPI≤4 (adjusted) (assessed with: PPI)** | | | | | | | | | |
| 2 | Phase 3 explanatory studies | not serious | not serious | not serious | not serious | strong association^c^, dose response gradient | 539 | HR = 2.04 (1.30-3.21) | ⨁⨁⨁⨁ High |
| **PPI≥6 vs PPI<6 (adjusted) (assessed with: PPI)** | | | | | | | | | |
| 3 | Phase 3 explanatory studies | serious^a^ | serious^e^ | not serious | not serious | strong association^c^, dose response gradient | 333 | HR = 2.52 (1.39-4.58) | ⨁⨁⨁◯ Moderate |
| **PPI as continuous variable (unadjusted) (assessed with: PPI)** | | | | | | | | | |
| 4 | Phase 3 explanatory studies | serious^a^ | serious^e^ | not serious | not serious | none | 815 | HR = 1.30 (1.22-1.38) | ⨁⨁◯◯ Low |
| **PPI≤4 vs PPI>6 (inpatient death) (unadjusted) (assessed with: PPI)** | | | | | | | | | |
| 2 | Phase 3 explanatory studies | serious^a^ | not serious | not serious | not serious | strong association,  dose response gradient | 274 | RR = 3.48 (2.46-4.91) | ⨁⨁⨁⨁ High |

#### Abbreviations: PPI (Palliative Prognostic Index), HR (hazard ratio), RR (risk ratio), CI (confidence interval)

#### Explanations

#### a. 1 study/domain was at high risk of bias

#### b. Both studies were at high risk of bias and had multiple domains with high risk of bias.

#### c. Strong association was defined as HR or RR>2 (GRADE Handbook, 2013)

#### d. Very strong association was defined as HR or RR>5 (GRADE Handbook, 2013)

#### e. Significant heterogeneity
